# *Plasmodium vivax* GPI-anchored micronemal antigen (PvGAMA) binds human erythrocytes independent of Duffy antigen status

**DOI:** 10.1038/srep35581

**Published:** 2016-10-19

**Authors:** Yang Cheng, Feng Lu, Bo Wang, Jian Li, Jin-Hee Han, Daisuke Ito, Deok-Hoon Kong, Lubin Jiang, Jian Wu, Kwon-Soo Ha, Eizo Takashima, Jetsumon Sattabongkot, Jun Cao, Myat Htut Nyunt, Myat Phone Kyaw, Sanjay A. Desai, Louis H. Miller, Takafumi Tsuboi, Eun-Taek Han

**Affiliations:** 1 Department of Medical Environmental Biology and Tropical Medicine, School of Medicine, Kangwon National University, Chuncheon, Gangwon-do 200-701, Republic of Korea; 2 Department of Parasitology, Wuxi Medical School, Jiangnan University, Wuxi, Jiangsu 214122, China; 3 Laboratory of Malaria and Vector Research (LMVR), National Institute of Allergy and Infectious Diseases (NIAID), National Institutes of Health (NIH), Rockville, MD 20852, USA; 4 Jiangsu Institute of Parasitic Diseases, Wuxi, Jiangsu, People’s China; 5 Department of Clinical Laboratory, The First Affiliated Hospital of Anhui Medical University, Hefei, Anhui, China; 6 Department of Parasitology, College of Basic Medicine, Hubei University of Medicine, Shiyan, Hubei, China; 7 Division of Malaria Research, Proteo-Science Center, Ehime University, Matsuyama, Ehime 790-8577, Japan; 8 Department of Molecular and Cellular Biochemistry, School of Medicine, Kangwon National University, Chuncheon, Gangwon-do 200-701, Republic of Korea; 9 Key Laboratory of Molecular Virology and Immunology, Unit of Human Parasite Molecular and Cell Biology, Institut Pasteur of Shanghai, Shanghai 200031, China; 10 Mahidol Vivax Research Unit, Faculty of Tropical Medicine, Mahidol University, Bangkok 10400, Thailand; 11 Department of Medical Research, Yangon, Myanmar

## Abstract

*Plasmodium vivax*, a major agent of malaria in both temperate and tropical climates, has been thought to be unable to infect humans lacking the Duffy (Fy) blood group antigen because this receptor is critical for erythrocyte invasion. Recent surveys in various endemic regions, however, have reported *P. vivax* infections in Duffy-negative individuals, suggesting that the parasite may utilize alternative receptor-ligand pairs to complete the erythrocyte invasion. Here, we identified and characterized a novel parasite ligand, *Plasmodium vivax* GPI-anchored micronemal antigen (PvGAMA), that bound human erythrocytes regardless of Duffy antigen status. PvGAMA was localized at the microneme in the mature schizont-stage parasites. The antibodies against PvGAMA fragments inhibited PvGAMA binding to erythrocytes in a dose-dependent manner. The erythrocyte-specific binding activities of PvGAMA were significantly reduced by chymotrypsin treatment. Thus, PvGAMA may be an adhesion molecule for the invasion of Duffy-positive and -negative human erythrocytes.

The interaction of *Plasmodium vivax* Duffy binding protein (PvDBP) with the Duffy blood group antigen on erythrocytes is thought to be the only ligand-receptor interaction that determines a failure to infect Duffy-null individuals^1^,^2^. As a micronemal protein, PvDBP releases to bind the Duffy antigen receptor for chemokines (DARC) on erythrocytes for processing invasion^3^,^4^,^5^. However, increasingly, vivax malaria has been observed in Duffy-negative blood group people^6^,^7^,^8^. *P. knowlesi* DBPα knockout mutants were able to interact with Duffy-positive human erythrocytes, but interactions with Duffy-negative cells was not evaluated^9^. Above all, we are looking for a novel molecular of *P. vivax* that could bind Duffy-null blood.

As an important erythrocyte-binding ligand, PvDBP was mapped to a Cys-rich region known as region II (PvDBPII) that is critical for erythrocyte receptor recognition^10^,^11^,^12^,^13^. As one of the leading *P. vivax* vaccine candidates, PvDBP is at the preclinical stage of vaccine development^14^,^15^. In addition to PvDBP, a large family of related proteins, termed *P. vivax* reticulocyte binding proteins (PvRBPs), are thought to selectively bind reticulocytes and may play critical roles in host-cell selection and commitment^16^,^17^,^18^,^19^. These PvRBPs are also being considered for vaccine development^20^ and as serological markers for diagnosis^21^. In contrast to a large collection of receptor-ligand pairs identified for human erythrocyte invasion by *P. falciparum*^22^,^23^, this limited repertoire of ligands identified to date in *P. vivax* has led some workers to suggest a lower level of redundancy for the invasion machinery of the parasite. Nevertheless, the existence of alternative invasion pathways for *P. vivax* remains controversial, with both computational analysis and experimentation suggesting a few different candidates^24^,^25^,^26^. Along with the clinical observation of *P. vivax* malaria in Duffy null patients, these observations suggest that Duffy-independent pathways may exist and deserve better exploration^8^.

An important group of antigens on invasive merozoites, the glycosylphosphatidylinositol-anchored proteins (GPI-APs), warrant examination, as they localize to the merozoite membranes or apical organelles and are considered vaccine candidates and some are predicted to play roles in invasion^27^. In *P. vivax*, several GPI-APs (PvMSP-1, −4, −5, −8, and −10, and Pv12, Pv34, Pv38 and PvMSP1P) have been characterized as blood-stage vaccine candidates, as immune responses against some of these proteins may yield protection^28^. One such protein, termed GPI-anchored micronemal antigen in *P. falciparum* (PfGAMA), was identified as a new erythrocyte-binding protein^29^. Although antibodies generated using recombinant PfGAMA expressed in *Escherichia coli* failed to inhibit invasion^29^, antibodies produced with a PfGAMA fragment synthesized in a wheat germ cell-free system (WGCF) successfully inhibited *P. falciparum* invasion, possibly because the WGCF preserves conformation-specific epitopes. Importantly, the erythrocyte binding epitope was mapped to the C-terminal region of PfGAMA. In addition, immune sera from animals revealed antibodies that react with PfGAMA and block invasion^30^, suggesting that this protein is a novel blood-stage vaccine candidate. However, *P. vivax* GAMA, the orthologue of PfGAMA, remains unstudied, particularly as a binding ligand for Duffy-positive or -negative erythrocytes. Therefore, in this study, we attempted to test our hypothesis that PvGAMA antigen as a ligand can bind to both Duffy-positive and/or -negative erythrocytes in a chymotrypsin-sensitive manner and characterize binding specificity with both specific antibodies and immune sera for blocking this process.

## Results

### Schematic of the primary structure of PvGAMA

**Figure 1:**
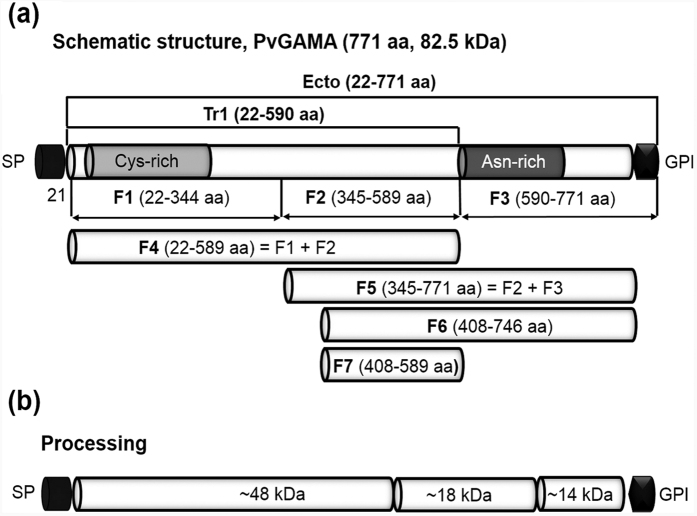
Schematic diagram of PvGAMA, fragments of PvGAMA for recombinant protein expression, erythrocyte binding assay and processing of PvGAMA. (**a**) Schematic diagram of PvGAMA. The PvGAMA protein comprises 771 amino acids with a calculated molecular mass of 82.5 kDa. Two regions of PvGAMA, PvGAMA-Ecto (Ecto; amino acid (aa) position 22–771) and -Tr1 (Tr1; aa 22-590), were designed for protein expression, and 7 fragments (F1, aa 22–344; F2, aa 345–589; F3, aa 590–771; F4, aa 22–589; F5, aa 345–771; F6, aa 408–746 and F7, aa 408–589) were designed for the erythrocyte binding assay. Indicated are the predicted signal peptide (SP; aa position 1–21) and glycosylphosphatidylinositol-anchor signal (GPI; aa 747–771). PvGAMA-Ecto and -Tr1 were used to raise specific antisera. (**b**) Processing of PvGAMA was predicted from western blot results of the parasite lysate probed with anti-PvGAMA-Ecto sera (Fig. 2c). aa, amino acid; kDa, kilodalton; SP, signal peptide; GPI, glycophosphatidylinositol-anchor signal; Cys, Cysteine; Asn, Asparagine.

**Figure 2:**
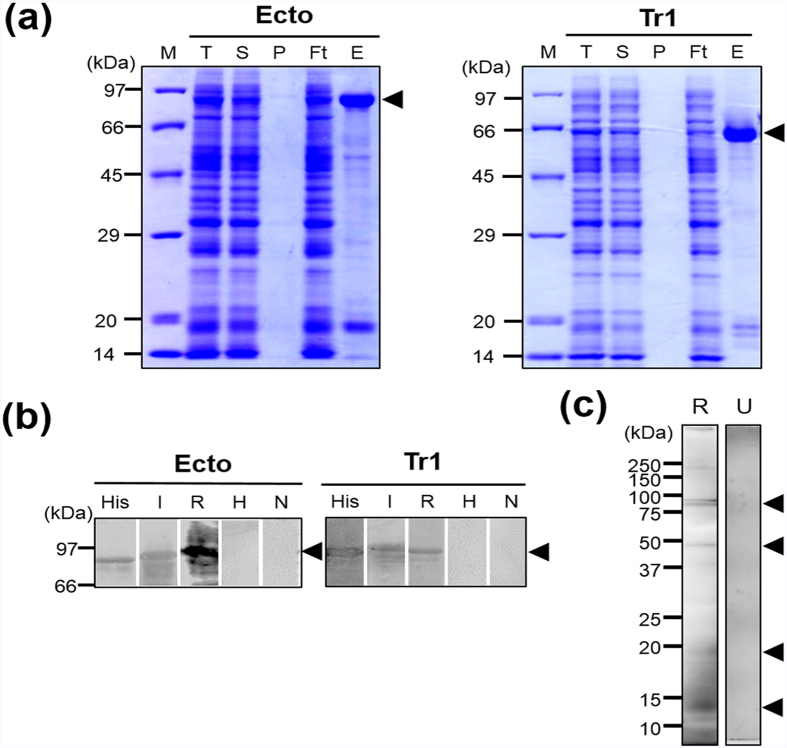
Recombinant protein expression, purification, and analysis of PvGAMA fragments. (**a**) PvGAMA-Ecto and PvGAMA-Tr1 were synthesized using the wheat germ cell-free protein expression system and purified on Ni-Sepharose columns. The purified fragments of PvGAMA were found in the soluble elution fractions. The arrowheads indicate specific bands for each recombinant protein. T, total translation mix; S, supernatant; P, pellet; Ft, flow through; E, elution. (**b**) The purified PvGAMA-Ecto and PvGAMA-Tr1 were resolved by SDS-PAGE, transferred to a PVDF membrane, and probed with an anti-His-tag antibody (His), vivax-infected human sera (I), or rabbit PvGAMA-Ecto immunized sera (R). Healthy human sera (H) and non-immunized rabbit sera were used as negative controls (N). (**c**) Schizont lysates under reducing condition probed with PvGAMA-Ecto immune sera (R). A pre-immunized rabbit serum was used as a negative control (U). The arrowheads indicate specific bands for each recombinant protein.

*Pvgama* gene sequence information encoded by PVX_088910 on chromosome 5 revealed that PvGAMA is a 771 amino acid protein with a predicted molecular mass of 82.5 kDa (Fig. 1a). The PvGAMA protein is encoded by a single exon gene and includes a signal peptide (aa 1–21), a GPI-anchor (aa 747–771), a cysteine-rich region (aa 54–220), and an asparagine(Asn)-rich region (aa 590–693). For the erythrocyte-binding assay, PvGAMA was divided into seven regions, from F1 to F7 (Fig. 1a). For immunization, we constructed PvGAMA-Ecto and PvGAMA-Tr1, and the two recombinant proteins were expressed using the WGCF system. A model for the proteolytic processing of PvGAMA (Fig. 1b) was consistent with the results presented in Fig. 2c. Based on the BLOSUM matrix, the amino acid sequence identity/similarity of PvGAMA and PfGAMA was 50.3/79.1% (Supplementary Fig. S1.). A conserved region (green, Supplementary Fig. S1.) is located at the C-terminus of the PvGAMA Asn-rich region (grey), but at the C-terminus of the PfGAMA alanine (Ala)/Asn-rich region (red), which shows erythrocyte binding ability.

### Expression of recombinant PvGAMA-Ecto and -Tr1 proteins, production of immune sera, antigenicity analysis, and proteolytic processing of PvGAMA

SDS-PAGE showed that all the recombinant proteins were expressed and purified from the soluble supernatant fraction (Fig. 2a). The PvGAMA-Ecto and -Tr1 fragments were expressed as predicted from their theoretical molecular weights as approximately 80 kDa and 65 kDa proteins, respectively. The PvGAMA-Ecto and -Tr1 were used to immunize rabbits to produce polyclonal antibodies. The corresponding immunoblots probed with an anti-His tag antibody, vivax-infected patient sera, or anti-PvGAMA-Ecto and -Tr1 rabbit sera, respectively, revealed similar and specific patterns of migration for each antigen (Fig. 2b), which proved that these recombinant proteins were antigenic to humans. On the other hand, malaria-naïve human sera samples and pre-immune rabbit sera samples were used as negative controls and did not react with the two target recombinant protein antigens (Fig. 2b). Western blot analysis of the schizont lysate probed with anti-PvGAMA-Ecto antisera and pre-immune rabbit sera showed that PvGAMA undergoes proteolytic processing events to form 14-, 18-, 48-, 80-, and 85-kDa products (Fig. 2c).

### Humoral immune response against PvGAMA

**Figure 3:**
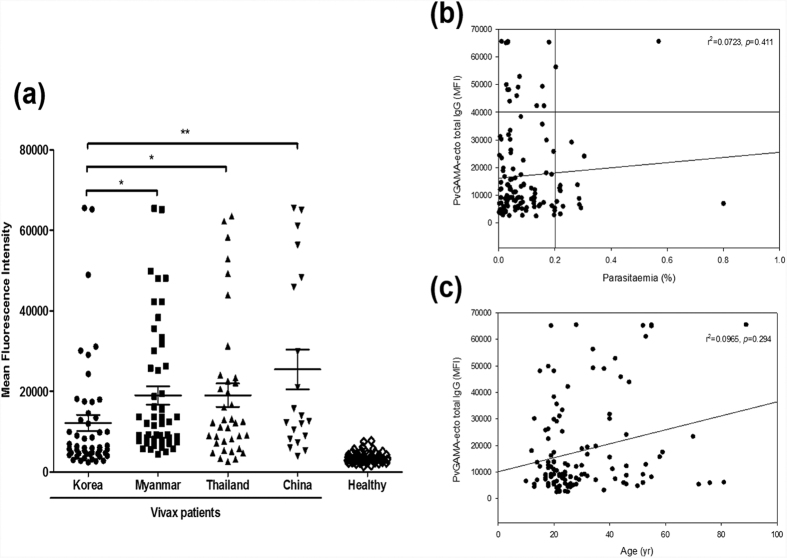
Natural acquired antibodies against the PvGAMA-Ecto antigen. (**a**) Total IgG prevalence of PvGAMA-Ecto with the *P. vivax* patient and healthy individual sera samples. The bar indicates the mean ± standard error of the fluorescence intensity. The *p*-values were calculated using Student’s *t*-test. (**b**) The correlation between PvGAMA-Ecto with parasitaemia was evaluated using Spearman’s correlation test. An MFI >40,000 was considered high intensity and parasitaemia >0.2% was considered high parasitaemia. (**c**) The correlation between PvGAMA-Ecto and parasitaemia was evaluated using Spearman’s correlation test. MFI, mean fluorescence intensity.

The antigenicity of PvGAMA-Ecto was evaluated in sera obtained from vivax malaria patients in malaria-endemic countries and healthy individuals in malaria-nonendemic areas in Korea. The prevalence of total IgG for the PvGAMA-Ecto in Korea, Myanmar, Thailand and China was 46.0%, 88.0%, 78.4%, 85.0%, and 72.0%, respectively (Table 1; Fig. 3a). The prevalences of total IgG in three countries, Myanmar, Thailand and China were significantly higher than in Korea. Significant differences were observed in the total IgG prevalence for the PvGAMA-Ecto between the vivax patients and healthy individuals (*p* < 0.0001) (Table 1; Fig. 3a). There was no significant correlation between parasitaemia and the humoral immune response (*r*^2^ = 0.0723, *p* = 0.411) or age (*r*^2^ = 0.0965, *p* = 0.294) to PvGAMA-ecto (Fig. 3b,c).

### PvGAMA colocalizes with the merozoite microneme marker PvDBP

**Figure 4:**
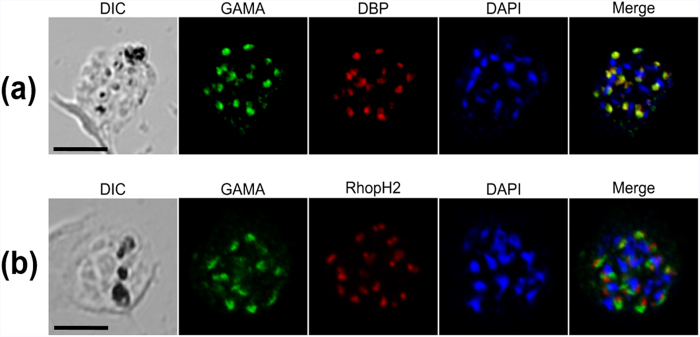
Subcellular localization of PvGAMA protein in asexual blood-stage parasites of *P. vivax*. (**a**) The acetone-fixed mature schizont of *P. vivax* was dual-labelled with rabbit immune sera against PvGAMA (green) and mouse immune sera against PvDBP (red). (**b**) Another mature schizont of *P. vivax* was also dual-labelled with rabbit immune sera against PvGAMA (green) and mouse immune sera against PvRhopH2 (PVX_099930, red). The nuclei are visualized with DAPI (blue); the bar represents 5 μm.

To determine the localization of the native PvGAMA protein in *P. vivax* merozoite, immunofluorescence assay was carried out using anti-PvGAMA and anti-PvDBP as microneme markers and anti-PvRhopH2 as a rhoptry marker on the mature schizont stage of *P. vivax*. The IFA results showed that the subcellular localization of PvGAMA detected using anti-PvGAMA antisera was colocalized with PvDBP (Fig. 4a), but differed from RhopH2 (Fig. 4b). These results suggested that the PvGAMA localizes to the microneme of the merozoite.

### Erythrocyte-binding activity of PvGAMA

**Figure 5:**
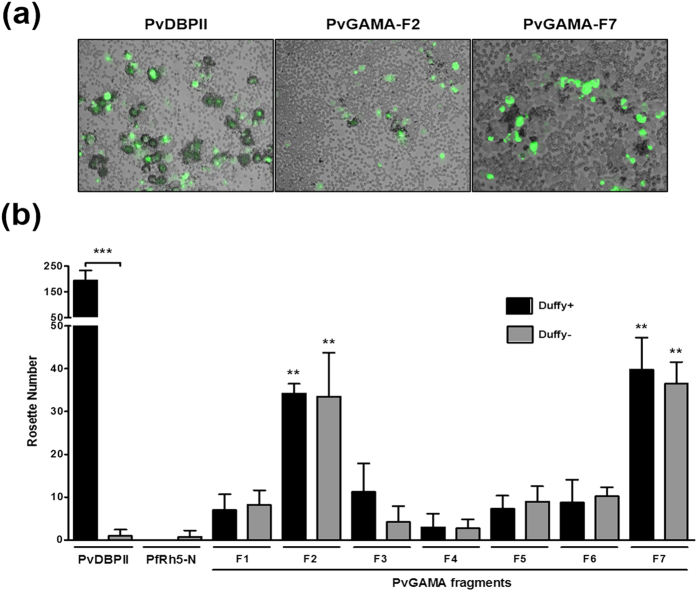
Binding specificity of PvGAMA fragments expressed on HEK 293T cells to Duffy-positive and -negative erythrocytes. (**a**) Erythrocyte-binding rosettes formed on the surface of HEK 293T cells expressing PvDBPII or different fragments of PvGAMA were visualized under light microscopy. (**b**) The number of rosettes formed by the HEK 293T cells transfected with genes coding for either PvDBPII, the non-binding domain of PfRH5 (PfRH5-N) or different fragments of PvGAMA (see Fig. 1a). Detection of the transfection efficiency of all constructs into HEK 293T cells by counting green signalling cells within 30 microscope fields (×200 magnification). Positive was defined as more than half the surface of the transfected cells covered with attached erythrocytes, and the total number of HEK 293T cells per coverslip was recorded. The data are shown as the mean number of rosettes of four independent experiments at different days, and the error bar represents ± standard deviation. The *p*-values were calculated using Student’s *t*-test. Significant differences are shown as double asterisks, *p* < 0.01, and triple asterisks, *p* < 0.0001.

The colocalization of PvDBP and PvGAMA suggests that PvGAMA may play an important role during parasite invasion of the erythrocyte. To elucidate the function of PvGAMA, the erythrocyte-binding activity of recombinant PvGAMA proteins was evaluated. HEK 293T cell transfection was assessed by detecting the expression of the green fluorescent protein (Supplementary Fig. S2, GFP) and anti-PvGAMA-Ecto immune sera (Supplementary Fig. S2, PvGAMA) with fluorescence microscopy. The transfection efficiency of each fragment was more than 80% by counting green fluorescent cells, and the positive rosettes were defined as adherent erythrocytes covering more than 50% of the HEK 293T cell surface (Fig. 5a). To confirm whether these PvGAMA fragments can bind to Duffy-positive and -negative erythrocytes, we counted the number of rosettes on HEK 293T cells transfected with each PvGAMA fragment. In the Duffy-positive blood group (Fig. 5b, black bar), all PvGAMA fragments had rosetting activity. Among them, PvGAMA-F2 (32 ± 3, mean of rosettes ± SD) and PvGAMA–F7 (40 ± 8) showed stronger rosetting activity than the other GAMA fragments. The erythrocyte rosetting ability of PvDBPII (195 ± 38) was significantly higher than that of all PvGAMA fragments (*p* < 0.001) (Fig. 5). In the Duffy-negative blood group (Fig. 5b, grey bar), all PvGAMA fragments except for PvGAMA-F3 or F4 showed rosetting activity (*p* < 0.05). PvGAMA-F2 (34 ± 10, *p* < 0.01) and PvGAMA-F7 (36 ± 5, *p* < 0.01) had higher rosetting activity than the other fragments. There was no difference between F2 and F7 in Duffy-positive and -negative erythrocyte rosetting ability. As a control, PvDBPII does not bind with Duffy-negative erythrocytes. The domain of PfRh5 that does not bind erythrocytes (PfRh5-N) was transfected and used in a binding assay as a negative control (Fig. 5b, control).

### Binding inhibition activity of antibodies on the erythrocyte binding of PvGAMA

**Figure 6:**
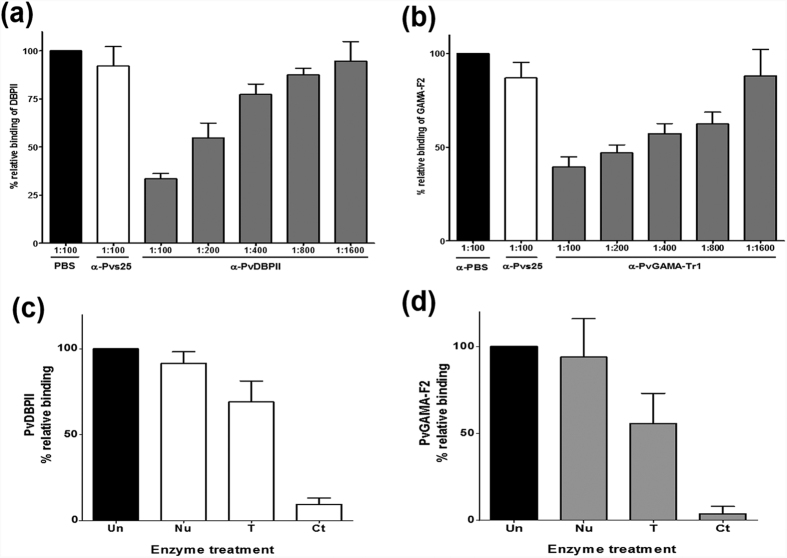
Inhibition of erythrocyte rosettes to PvGAMA-F2 by anti-PvGAMA-Tr1 antibody and receptor specificities. (**a**) HEK 293T cells were transfected with pEGFP-HSVgD1_DBPII plasmid DNA expressing a GFP-DBPII fusion protein. The cells were subsequently incubated with anti-PvDBPII rabbit sera at various dilutions before the addition of human erythrocytes. Binding was scored by counting the number of rosettes bound to HEK 293T cells in 30 microscope fields (×200 magnification). Rabbits immunized with PBS as a non-immunized control (PBS) and anti-Pvs25 (no erythrocyte-binding activity) immune rabbit sera diluted 1:100 were included as negative controls (Pvs25). (**b**) Inhibition of erythrocyte binding to PvGAMA-F2 expressed on HEK 293T cells by PvGAMA-Tr1 immune rabbit sera. The cells were incubated with PvGAMA-Tr1 immune rabbit sera at various dilutions before the addition of human erythrocytes. Error bars represent ± standard deviations. The erythrocyte binding abilities of HEK 293T cells transfected with pEGFP-HSVgD1_PvDBPII and PvGAMA-F2 plasmid DNA expressing a GFP-PvDBPII (**c**) and GFP-PvGAMA-F2 (**d**) were tested by incubation with untreated (Un), neuraminidase-treated (Nu), trypsin-treated (T), and chymotrypsin-treated (Ct) erythrocytes. The bars represent the standard deviation of the means of the three independent experiments.

An *in vitro* binding inhibition assay was used to determine whether the antibody response correlated with an inhibitory effect of human sera against PvGAMA-Ecto and PvGAMA-Tr1 cytoadherence activity. A PBS-immunized rabbit sera and rabbit sera against Pvs25 (PVX_111175), the ookinete surface protein (Fig. 6), were used as negative controls^31^. To ensure the properties and validity of our assay, we included PvDBPII as a control and performed an erythrocyte binding inhibition assay using PvDBPII-transfected HEK 293T cells and serially diluted anti-PvDBPII sera (Fig. 6a). The difference in the inhibitory efficacies of these antisera may reflect the difference in their antibody titres against the binding epitope(s). The rabbit anti-PvGAMA-Tr1 antibody had a dose-dependent inhibitory effect on the *in vitro* binding of human erythrocytes to the transfected HEK 293T cells expressing the PvGAMA-F2 (Fig. 6b). Comparatively, there was not a strong inhibitory relationship between the anti-PvGAMA-Ecto antisera and the PvGAMA-F2 protein, although weakly inhibitory activity was detected using these antisera (data not shown).

### Receptor specificity for PvGAMA

The erythrocyte-binding specificity of PvDBPII and PvGAMA-F2 was studied by testing the binding to enzyme-treated erythrocytes (Fig. 6c,d). Neuraminidase treatment of erythrocytes removes sialic acid (SA) residues in SA–containing erythrocyte receptors, and trypsin or chymotrypsin treatment differentially cleaves the peptide backbones of erythrocyte receptors. Neuraminidase-treated erythrocytes bound to PvGAMA-F2 (mean value of relative binding, 93.9%) transfected HEK 293T cells as strongly as did untreated erythrocytes. Although trypsin-treated erythrocytes appeared to bind slightly to PvGAMA-F2 (55.8%) transfected HEK 293T cells, chymotrypsin-treated erythrocytes failed to bind to PvGAMA-F2 (3.9%) transfected HEK 293T cells. The binding of native PvGAMA was resistant to neuraminidase treatment and partially resistant to trypsin treatment, but strongly sensitive to chymotrypsin treatment. As a control for enzyme treatments, PvDBPII was also examined. As expected, PvDBPII bound to erythrocytes in a chymotrypsin-sensitive, trypsin-resistant and neuraminidase-resistant manner. Taken together, these results indicate that PvGAMA binds a nonsialylated protein receptor.

## Discussion

The complex processes of the *P. vivax* life cycle act as a barrier for discovering new vaccine candidates and drug targets. We are particularly interested in identifying a protein that may be involved in the basic machinery of the host cell invasion. Several antigens have been identified for normocyte or reticulocyte binding ability from *P. vivax*. These antigens include the PvDBP and PvRBP family^16^,^19^ on the microneme and PvMSP1 and PvMSP1P^26^ on the merozoite surface. However, complex processes in reticulocyte and/or Duffy-negative-specific invasion mechanisms of simultaneous occurrence were insufficient in *P. vivax*. This study was performed with the objective of characterizing PvGAMA, the homologue of PfGAMA, showing not only Duffy-positive but also Duffy-negative erythrocyte binding activities as one of ligand candidates for erythrocyte binding or invasion of *P. vivax* parasites.

In a recent report, associations between antibodies to merozoites antigens and protection from episodes of symptomatic malaria were examined in a longitudinal cohort study. An antibody against PfGAMA showed an intermediate level of protection^32^. It may have partial protective associations from high antibody response combinations together with other antigen candidates. In parallel, of the highly immunoreactive antigens, the IgG antibody level against PvGAMA was much higher with age, reflecting cumulative life time exposure, than with current infection^32^,^33^ and was significantly higher in exposed cases^34^. We suggest that these IgG prevalence differences may be due to exposure frequency to malaria in high endemic areas; however, this may require further study.

In the *P. vivax* parasite life cycle, only a few proteins have been identified as erythrocyte-binding ligands during invasion. Transcriptomic analysis of blood-stage vivax parasites (PlasmoDB) shows that PvGAMA is highly upregulated in the schizont stage parasite. There is commonality among all *Plasmodium* species, including primary molecular structure, stage-specific expression profile, and high conservation, suggesting that the function of PvGAMA may be as important as other species of GAMA during parasitic invasion of erythrocytes. Previous findings suggested that primary and secondary processing events occur in PfGAMA^29^ and secondary processing occurs ‘prior to invasion’. In addition, the detection of processed fragments (~14-, 18- and 48-kDa) of PvGAMA in the parasite lysate (Fig. 1b) demonstrates that PvGAMA proteolytic cleavage occurs in a manner similar to that for PfGAMA from the merozoite surface. However, the fragments will be required for further study.

The most important ligand, PvDBP, has been identified as an essential erythrocyte-binding protein, which interacts with the Duffy antigen on the erythrocyte membrane and is required for tight junctions and invasion of human erythrocytes^2^,^3^,^35^. With more reports on frequent transmission in the Duffy-negative population and as novel erythrocyte-binding ligands of *P. vivax* have been identified, the invasive pathway of *P. vivax* is becoming more and more complex. Recently, of the PvRBP family proteins, PvRBP1a and 1b were reported to have reticulocyte-specific binding activity with chymotrypsin-sensitive receptor^16^, and PvRBP2a was also identified to have binding activity to RBCs with the trypsin-sensitive receptor^19^. However, these observations are insufficient for explaining the invasion of Duffy-negative host cells by *P. vivax*. Therefore, we hope to identify molecules involved in alternative pathways in *P. vivax* invasion. From our immunofluorescence analyses, it appears likely that PvGAMA is a micronemal protein of merozoites, according to PvGAMA colocalizing with PvDBP but not with rhoptry protein PvRhopH2 (Fig. 3). As introduced above, together with the GPI-anchor, features indicate PvGAMA may play some roles during erythrocyte invasion. One of the apicomplexan parasites, *Toxoplasma gondii* GAMA (TgGAMA) also has conserved antigens and an essential role in adhesion to the cells^36^. TgGAMA included a conserved N-terminal region with PfGAMA and localized at the microneme of *T. gondii*, likely PvGAMA^36^. As the homologue of PvGAMA, the PfGAMA C-terminal section has been identified as an erythrocyte-binding protein^30^. Compared to PfGAMA, our results show that PvGAMA protein binds with both Duffy-positive and -negative erythrocytes (Fig. 5). Interestingly, fragments 2 and 7 of PvGAMA contained a conserved domain with PfGAMA for erythrocyte binding. To further analyse the inhibitory activity of specific antibodies induced by recombinant PvGAMA proteins, the anti-PvGAMA-Tr1 antibodies possessed strong inhibitory effects on PvGAMA-F2 protein erythrocyte binding at a 1:100 or lower (i.e., < 1:100) dilution (Fig. 6b). This result indicates that PvGAMA-F2 and/or –F7 may be important domains for erythrocyte binding interactions as well as invasion. From the enzyme specificity of the erythrocyte surface protein, PvGAMA reacted with a novel SA-independent receptor, and it shared the known binding profile of PfGAMA and PvDBP. This result strongly suggests that PvGAMA may play a key role as a specific receptor for binding and invasion to erythrocytes.

In summary, PvGAMA is a novel micronemal antigen of *P. vivax* merozoite that binds to Duffy-positive and -negative erythrocytes. Duffy-negative erythrocyte binding of this parasite molecule may explain the occurrence of Duffy-negative blood group patients infected by *P. vivax*. Erythrocyte binding assays showed that PvGAMA possesses an erythrocyte-binding epitope, and it binds a nonsialylated protein receptor. Anti-PvGAMA antibodies specifically block protein erythrocyte binding. Although infected Duffy-negative individuals may express some special molecules that allow *P. vivax* invasion, our findings provide another possible pathway for the *P. vivax* invasion of erythrocytes.

## Materials and Methods

### Human blood and sera samples

The sera from the vivax malaria patients were collected from people with symptoms and positive vivax parasitaemia by microscopic examination at local health centres and clinics in four different countries: Gangwon Province in the Republic of Korea (ROK), Shwegyin in Myanmar, Kanchanaburi in Thailand, and Anhui in China (Table 2). The sera of healthy individuals were collected from malaria-naïve people living in nonendemic areas of the ROK. Duffy-positive and -negative blood was supplied by the NIH blood bank. Blood was collected in 10% (vol/vol) citrate phosphate dextrose and stored for up to 4 weeks at 4 °C. At the time of the study, the erythrocytes were washed three times in incomplete Dulbecco’s modified Eagle’s medium (incomplete DMEM; Invitrogen, Carlsbad, CA, USA). All experiments were performed in accordance with relevant guidelines and regulations, and all experimental protocols involving human samples were approved by the Ethical Committees from the Kangwon National University Hospital, ROK, the Department of Medical Research, Myanmar, the Faculty of Tropical Medicine, Mahidol University, and Jiangsu Institute of Parasitic Diseases, China. Written informed consent was obtained from all subjects.

### Expression of recombinant PvGAMA proteins

The primers for PvGAMA fragments were designed based on PvGAMA of the *P. vivax* Sal-1 strain sequence (PlasmoDB PVX_088910) and used for amplification of PvGAMA fragments from genomic DNA of *P. vivax* isolates from the ROK. Different truncated versions of PvGAMA proteins were synthesized and used for raising antibodies (Fig. 1). Briefly, the PVX_088910 fragments encoding ECTO [denotes full-length GAMA without signal peptide and comprising aa 22 to aa 771 and a hexahistidine (His) tag at the C-terminus] and Tr1 (denotes truncate 1 and comprising aa 24 to aa 590 and a His tag at C- terminus) were amplified using sense primers with XhoI sites and antisense primers with BamHI restriction sites (shown in small letters in the primer sequences below). The primer pairs Ectof (5′-GGGCGGATATctcgagATACGGAATGGAAACAACCC-3′) and Ector (5′- GCGGTACCCGggatccTTAAAAAATGAATAGGAGCAACGC-3′), and Tr1f (5′-ATCACTAGTTctcgagAATGGAAACAACCCGCAG-3′) and Tr1r (5′- CCCTATATATggatccTCAGTGAGTGATGATGATGATGATGGTTTCCGCTCCCGTTGAC-3′) were used to generate the DNA fragments encoding the Ecto and Tr1 proteins, respectively. The underlined sequences in the above primers indicate the region that encodes His tags. The amplified fragments were then restricted and ligated into the wheat germ cell-free expression vector pEU-E01-His-TEV-MCS (CellFree Sciences, Matsuyama, Japan). The cloned inserts were sequenced using an ABI 3700 Genetic Analyzer (Genotech, Daejeon, Korea). The recombinant proteins with His-tags were expressed using a wheat germ cell-free system (CellFree Sciences) and purified using a Nickel-Sepharose column (GE Healthcare Life Sciences, Uppsala, Sweden) as previously described^37^.

### Production of animal immune sera

To generate antibodies against PvGAMA-Ecto and PvGAMA-Tr1 in rabbits, one Japanese red rabbit was immunized subcutaneously with 250 μg of purified proteins with Freund’s complete adjuvant (Sigma-Aldrich, St. Louis, MO, USA), followed by 250 μg of Freund’s incomplete adjuvant (Sigma-Aldrich) as previously described^30^. All immunizations were performed 3 times at 3-week intervals. The antisera were collected 2 weeks after the final boost. Anti-PvDBPII and –RhopH2 mouse or rabbit antibodies were described in our previous studies^26^,^38^. All animal experimental protocols were approved by the Institutional Animal Care and Use Committee of Ehime University, and the experiments were conducted according to the Ethical Guidelines for Animal Experiments of Ehime University.

### SDS-PAGE and western blot analysis

A total of 10 μg of each recombinant PvGAMA protein was prepared in reducing sample buffer, separated by 12% SDS-PAGE, and stained with Coomassie brilliant blue. *P. vivax* parasites rich in schizonts were harvested from 10 ml of a patient blood sample (parasitaemia >0.1%) using the Percoll-gradient method, and the parasite proteins were extracted in SDS-PAGE loading buffer. One-tenth of the parasite lysate was loaded in each lane and separated by 12% SDS-PAGE. For western blot analysis, the proteins were transferred electrophoretically to PVDF membranes (Millipore Corp., Bedford, MA, USA) and incubated with blocking buffer (5% nonfat milk in PBS containing 0.2% Tween 20, PBS/T) for 1 h at 37 °C. The blots containing recombinant proteins were then incubated for 1 h at 37 °C with either anti-Penta-His antibody (QIAGEN, Hilden, Germany), anti-PvGAMA-Ecto, anti-PvGAMA-Tr1, pooled vivax infected patient sera, pre-immunized rabbit sera, or pooled non-infected human sera diluted 1:1000 in PBS/T. The membranes were washed with PBS/T and incubated with IRDye^®^ goat anti-rabbit or IRDye^®^ goat anti-human (LI-COR Bioscience, Lincoln, NE, USA) to detect recombinant proteins according to the manufacturer's instructions. The data were scanned using an Odyssey infrared imaging system (LI-COR Biosciences) and analysed with Odyssey software (LI-COR Bioscience).

### Protein arrays

To develop the protein arrays, sera from patients with vivax malaria and malaria-naïve individuals were used to analyse the humoral immune response by well-type amine arrays. A series of double dilutions was used to optimize the coating antigen concentration (0.1 to 200 ng/μl) of PvGAMA-Ecto. The optimized concentrations of the purified recombinant proteins per well of the array were determined as 50 ng/μl for PvGAMA-Ecto in PBS/T. The PvGAMA-Ecto spotted slides were then incubated for 1 h at 37 °C. Each well was blocked with 1 μl of blocking buffer (5% BSA in PBS/T) incubated for 1 h at 37 °C. The arrays also contained an area spotted with purified PvMSP1-19 (12.5 ng/μl in PBS/T) as a positive control and wheat germ lysate without any plasmid vector as a negative control. The chips were then probed with sera from the malaria patients or healthy individuals (1:25 dilution). Alexa Fluor 546-conjugated goat anti-human IgG (10 ng/μl, Invitrogen) in PBS/T was used as the detection antibody, and the fluorescent signals were scanned in a fluorescence scanner (InnoScan 300-G; Innopsys, Carbonne, France) and quantified. Array spot fluorescence intensity was analysed by MAPIX software version 7.4.1. (Innopsys), and the correlation of parasitaemia and age was calculated using SigmaPlot 12 (Systat Software Inc., San Jose, CA, USA). The cutoff value was defined as two standard deviations (SDs) above the mean fluorescence intensity of the negative control samples.

### Indirect immunofluorescence assay (IFA)

Immunofluorescence assays were performed on acetone-fixed parasites as previously described^39^. The following primary antibody dilutions were used: rabbit anti-PvGAMA-Ecto (1:100), mouse anti-PvDBPII (1:50), or mouse anti-PvRhopH2 (1:100). The following secondary antibodies were used: Alexa-568 goat anti-mouse IgG (1:500; Invitrogen), Alexa-488 goat anti-rabbit IgG (1:500; Invitrogen) or DAPI (4′,6′-diamidino-2-phenylindole) for nuclear staining (1:1,000; Invitrogen). The slides were mounted in ProLong Gold antifade reagent (Invitrogen) and visualized under oil immersion using a confocal scanning laser microscope (LSM710; Carl Zeiss MicroImaging, Thornwood, NY, USA) with a Plan-Apochromat 63 × /1.4 oil differential interference contrast (DIC) objective lens. The images were captured with Zen software (Carl Zeiss MicroImaging) and prepared for publication using Adobe Photoshop (Adobe Systems, San Jose, CA, USA).

### Erythrocyte-binding assay on the surface of transfected HEK 293T cells

The pEGFP-HSVgD1-N1 vector was used to construct HEK 293T expression vectors encoding PvGAMA-F1, F2, -F3 and PvDBPII as previously described^40^. Here, we prepared PvGAMA-F1, -F2, and -F3 fragments by PCR amplification with primers listed as follows: the primer pairs F1f (5′-ggtcctggacgaattcATACGGAATGGAAACAACCC-3′) and F1r (5′-gtgtatggggccttgggcccTCCTTGCGTAACTTCGCG-3′); F2f (5′-ggtcctggacgaattcGATGTGAGCGTTGATGAGAAGG-3′) and F2r (5′-gtgtatggggccttgggcccTCCGCTCCCGTTGACGC-3′); F3f (5′-ggtcctggacgaattcAACGCAGCAAATGCGAAC-3′) and F3r (5′-gtgtatggggccttgggcccAAAAATGAATAGGAGCAACGCA-3′); F4f was the same as F1f, and F4r was the same as F2r; F5f was the same as F2f, and F5r was the same as F3r; F6f (5′-ggtcctggacgaattcAACATGGGTGCCACCTCC-3′) and F6r (5′-gtgtatggggccttgggcccCTGCAATTCGAGGTTTTTAAAGG-3′); F7f was the same as F6f, and F7r (5′-gtgtatggggccttgggcccTCCGCTCCCGTTGACGC-3′). The underlined sequences in the above primers indicate the vector sequence. The primers were used to generate the DNA fragments encoding the F1, F2, F3, F4, F5, F6 and F7 proteins, respectively. Each amplified gene fragment in 2 μl of the PCR product was ligated with 100 ng of linearized pEGFP-HSVgD-N1 vector using the In-Fusion^®^ HD Cloning Kit (Clontech, Mountain View, CA, USA). These constructs are designated as pEGFP-PvGAMA-F1, -F2, -F3, -F4, -F5, -F6, and -F7 and were purified using an Ultrapure Plasmid Extraction System (Viogene, Taipei, Taiwan).

HEK 293T cells were plated into 24-well culture plates and transfected with each of the above pEGFP construct DNAs (100 ng per well) using Lipofectamine (Invitrogen) in sera-free incomplete DMEM. After a 42–44 h incubation, transfected HEK 293T cells were incubated with 250 μl of diluted antisera against each fragment of the target protein in incomplete DMEM at 37 °C for 1 h. After washing, the transfected cells were incubated for 2 h at 37 °C with human erythrocytes (blood group O^+^, 1% haematocrit in incomplete DMEM). To determine transfection efficiency, green fluorescence cells (GFP-tag) were observed unfixed on a Flowview^®^ FV1000 Laser Scanning Confocal Imaging System (Olympus, Tokyo, Japan) at a magnification of 200× to score. The images were analysed and prepared using Adobe Photoshop CS5. The cells were washed three times with PBS to remove the nonadherent erythrocytes, and the number of rosettes in 30 microscopic fields was counted (total number, 1500 ~ 2000 cells) at a magnification of 200× to score erythrocyte-binding activity. Positive rosettes were defined as adherent erythrocytes covering more than 50% of the HEK 293T cell surface^40^. The cells were counted unfixed using a light microscope (Olympus, Tokyo, Japan). Images were captured by eXcope software (DIXI Optics, Daejeon, Korea) and analysed and prepared using Adobe Photoshop CS5.

Enzyme treatments of erythrocytes were performed as previously described^30^,^41^. Briefly, sialic acid residues were removed by incubating 100 μl of packed human erythrocytes with neuraminidase (final concentration of 66.7 mU/ml in iRPMI) on a rotating wheel for 1 h at 37 °C. For trypsin or chymotrypsin treatments, 100 μl of packed human erythrocytes were incubated with trypsin or chymotrypsin (final concentration of 1 mg/ml in iRPMI) on a rotating wheel for 1 h at 37 °C and subsequently incubated with soybean trypsin inhibitor (final concentration of 0.5 mg/ml in iRPMI) for 10 min at 37 °C to inhibit the trypsin or chymotrypsin. After the enzyme treatments, the erythrocytes were washed twice with 10 ml of iRPMI, resuspended in iRPMI at a 50% haematocrit, stored at 4 °C and used within a week.

The experiments using each antiserum were performed in triplicate and repeated twice unless otherwise stated. Immune rabbit sera were first tested at a 1:100 (immune sera) dilution to assess their inhibitory activity. Serial dilutions for each antiserum were then tested to titrate the erythrocyte-binding inhibitory activity. The binding inhibitory activity for each antiserum dilution was compared with the binding inhibitory activity of the negative control sera (either a 1:100 dilution of non-immunized rabbit sera). The results are expressed as the relative percent binding activity (negative control sera = 100% binding activity).

### Fluorescence microscopy

Surface expression of each PvGAMA fragment on HEK 293T cells transfected with target plasmid DNA was detected using a 1:100 dilution of rabbit antisera against PvGAMA-Ecto or rabbit antisera against PvDBPII as the primary antibody and Alexa Fluor 568-conjugated goat anti-rabbit antibodies (Invitrogen) as the secondary antibody. The GFP-tag of each recombinant protein was detected using a 488-nm laser. The cells were observed unfixed on a Flowview^®^ FV1000 Laser Scanning Confocal Imaging System (Olympus, Tokyo, Japan). The images were analysed and manipulated using Adobe Photoshop CS5.

### Statistical analysis

The data were analysed using GraphPad Prism (GraphPad Software, San Diego, CA, USA). For protein array and erythrocyte-binding assays, Student’s *t*-test was used to compare the means of each group. A *P* value of < 0.05 was considered significant. Linear regression was used to create the standard curves.

## Additional Information

**How to cite this article**: Cheng, Y. *et al*. *Plasmodium vivax* GPI-anchored micronemal antigen (PvGAMA) binds human erythrocytes independent of Duffy antigen status. *Sci. Rep.* **6**, 35581; doi: 10.1038/srep35581 (2016).

## Supplementary Material

Supplementary Information

## Figures and Tables

**Table 1:** Prevalence, 95% confidence intervals, and mean fluorescence intensity of IgG responses to PvGAMA-Ecto fragment in vivax malaria patients and healthy individual serum samples.

Patients/healthy from each country	No. of samples					
	Positive	Negative	Total (%)^a^	95% CI^b^	MFI^c^	*p* value^e^
Patients						
Korea	23	27	50 (46.0)	32.9–59.6	12139.2	*p* < 0.0001
Myanmar	44	6	50 (88.0)	76.2–94.4	18986.9	*p* < 0.0001
Thailand	29	8	37 (78.4)	62.8–88.6	19039.2	*p* < 0.0001
China	17	3	20 (85.0)	64.0–94.8	25453.1	*p* < 0.0001
Total	113	44	157 (72.0)	64.5–78.4	17642.1	
Healthy individuals						
Korea	2	48	50 (96.0)	86.5–98.9	3540.2	

^a^Sensitivity: percentage of positive in patient samples.

^b^CI: confidence interval.

^c^MFI: mean fluorescence intensity.

^d^Specificity: percentage of negative in healthy samples.

^e^Differences in the total IgG prevalence for each antigen between vivax patients and healthy individuals were calculated by Student’s *t*-test. A *p* value of < 0.05 is considered statistically significant.

**Table 2:** Characteristics of study patient serum samples from endemic areas of Korea, Myanmar, Thailand and China and healthy individuals.

Acute vivax patients					Healthy subjects
Characteristic	Korea	Myanmar	Thailand	China	Korea
Total (*n*)	50	50	37	20	50
Age (year)					
Mean (SD^a^)	34.9 (21.6)	24.0 (8.5)	31.5 (12.5)	36.3 (16.5)	9.1 (2.0)
Range	12–89	10–52	18–70	17–58	6–13
Parasitaemia (%)					
Mean (SD)	0.102 (0.113)	0.896 (0.118)	0.099 (0.096)	0.087 (0.016)	
Range	0.002–0.570	0.009–0.801	0.006–0.305	0.009–0.204	

^a^SD: standard deviation.
