# Supplementary material for: Plasmodium vivax GPI-anchored micronemal antigen (PvGAMA) binds human erythrocytes independent of Duffy antigen status
Source: Sci Rep. 2016 Oct 19;6:35581. doi: 10.1038/srep35581 (PMC5069673; doi:10.1038/srep35581)
Supplement: Supplementary Information [file srep35581-s1.pdf]

Supplementary Information

Plasmodium vivax GPI-anchored micronemal antigen (PvGAMA) binds human erythrocytes independent of Duffy antigen status

Yang Cheng[1,2,3], Feng Lu[1,4], Bo Wang[1,5] , Jian Li[1,6], Jin-Hee Han[1], Daisuke Ito[3,7], Deok-Hoon Kong[8], Lubin Jiang[9], Jian Wu[3], Kwon-Soo Ha[5], Eizo Takashima[7], Jetsumon Sattabongkot[10], Jun Cao[4], Myat Htut Nyunt[1,11], Myat Phone Kyaw[11], Sanjay A. Desai[3], Louis H. Miller[3], Takafumi Tsuboi[7] & Eun-Taek Han[1]

```
PfGAMA   MKYYTSLYVALIIALCQAVSALIRNSNTPQAFLIPELNNNEKNEFNNNEKNEMNNNLNNE    60
PvGAMA   MKCNASLLVLLSALLSAANALIRNGNNPQALVPEKGADP--------------SGGQNNR    46
              .:  :.*: ** .*..*****.*.***::  :  :              ... **.

PfGAMA   FNNNEENCDIQKIAEEMMENLLNENDMYTNIMLSLQNRLSDDYLCSEPKYENICIHEKDK   120
PvGAMA   SGENQDTCEIQKMAEEMMEKMMKEKDVFSSIMEPLQSKLTDDHLCSKMKYTNICLHEKDK   106
           .:*::.*:***:******::::*:*:::.** .**.:*:**:***: ** ***:*****

PfGAMA   ISLSFPCSNPKYEKLIHKFTFKKLCNSKAAFNNTLLKSFIEEDEEQNTFSLMLKQFKILL   180
PvGAMA   TPLTFPCTSPQYEQLIHRFTYKKLCNSKVAFSNVLLKSFIDKKNEENTFNTIIQNYKVLS   166
           .*:***:.*:**:***:**:*******.**.*.******::.:*:***. :::::*:*

PfGAMA   TCVDDELKDIYKESIDLLVDLKTSITELTQKLWSGKMVNVLKKREFLITGILCELRNGNK   240
PvGAMA   TCIDDDLKDIYNASIELFSDIRTSVTEITEKLWSKNMIEVLKTREQTIAGILCELRNGNN   226
         **:**:*****: **:*: *::**:**:*:**** :*::***.**  *:**********:

PfGAMA   SPLISKSLEFENLGILKMNNEELLNEAYNAFSDYYYFFPYFIQKLLEKGGMIERLIKIHE   300
PvGAMA   SPLVSNSFSYENFGILKVNYEGLLNQAYAAFSDYYSYFPAFAISMLEKGGLVDRLVAIHE   286
         ***:*:*:.:**:****:* * ***:** ****** :** *  .:*****::**: ***

PfGAMA   NLTKYRTKDMVNKINAQSKGEVLNNEDILNKLNAYKHYTKHGATSFIQSREVKIVNQNVN   360
PvGAMA   SLTNYRTRNILKKINEKSKNEVLNNEEIMHSLSSYKHHAGGTRGAFLQSRDVREVTQGDV   346
         .**:***:::::*** :**.******:*::.*.:***::      :*:***:*: *.*.

PfGAMA   NDDTTKNQQQNVNNNEKLNNNNNNNNNQQVNNNNNNNNQQVNNNNNNNNNQVNNNNNNNN   420
PvGAMA   SVDEKGDRATTAGGNQSASVAAAAPKDAGPTVAAPNTAATLKTAASPNAAATNTAAPPNM   406
         . * . ::   ....*:. .        ::    .     *.    ::.  . *   .*.     *

PfGAMA   NQVNNNNYNNNNQVNNNNNNNQQVNNNNNYNNQLNNNNFNNNLQVNKNDKHVPKNNHTTA   480
PvGAMA   G-----------------------------------------------------------   407

PfGAMA   THTNNILYNPLYSINPEKPKDIIKLLKDLIKYLHIVKFENNEPTTNIDEEGIRKLLENSF   540
PvGAMA   --ATSPLSNPLYGTSSLQPKDVAVLVRDLLKNTNIIKFENNEPTSQMDDEEIKKLIESSF   465
         :.. * ****. .. :***:   *::**:*   :*:********:::*:* *:**:*.**

PfGAMA   FDLNDDILIVRLLLKPQTVILTVIQSFMLMTPSPSRDAKAYCKKALINDQLVPTNDTNIL   600
PvGAMA   FDLSDNTMLMRLLIKPQAAILLIIESFIMMTPSPTRDAKTYCKKALVNGQLIETSDLNAA   525
         ***.*: :::***:***:.** :*:**::*****:****:******:*.**: *.* *

PfGAMA   SEENELVNNFSTKYVLIYEKMKLQELKEMEESKLKMKYSKTNLSALQVTNPQNNKDKNDA   660
PvGAMA   TEEDDLINEFSSRYNLFYERLKLEELREIEQNRKALKNSKGTLSVLEVANSQNAPDGKGV   585
         :**::*:*:**::* *:**::**:**:*:*:.:  :* ** .**.*:*:*.**  *  :..

PfGAMA   S-----------------------------------------------------------   661
PvGAMA   NGSGNAANANAANANAANANAANANAANGNLANANLANANAANADAANANAANANAANAN   645

PfGAMA   --------------------------------------------------NKNNNPNNSST   672
PvGAMA   AANANLANANLANANLANANLANANLANANLANANAANANAANANAANGNAPNSNNGSGS   705
                                                           *   *.  *.*.:

PfGAMA   PLIAVVTDLSGEKTEDIINNNVDIATLSVGVQNTFQGPNAKAGSLINHLSYATFLFFSFI   732
PvGAMA   PLIVVVGADLGEKTEDIIKNNVDVAALTADVEQAFKNLELQSGSFSANLSHALVLLSSIA   765
         ***.**     ********:****:*:*:..*:::*:. : ::**:  :**:* .*: *:

PfGAMA   LINLLN                                                         738
PvGAMA   LLLFIF                                                         771
         *: ::
```

Supplementary Figure S1. Amino acid sequence alignment of PfGAMA and PvGAMA. Cys residues with yellow areas indicate Cys residues conserved among both sequences and those with dark areas

masks indicate conserved Asn residues region. High conserved region between PvGAMA and PfGAMA was marked with green. Blue line indicated PvGAMA-F2. Ala/Asn-riched region marked with red at the C-terminus of PfGAMA. Dashes indicate a deletion. Asterisks, colons and dots under the alignment indicated identical, conserved, and semi-conserved substitutions, respectively, based on BLOSUM.

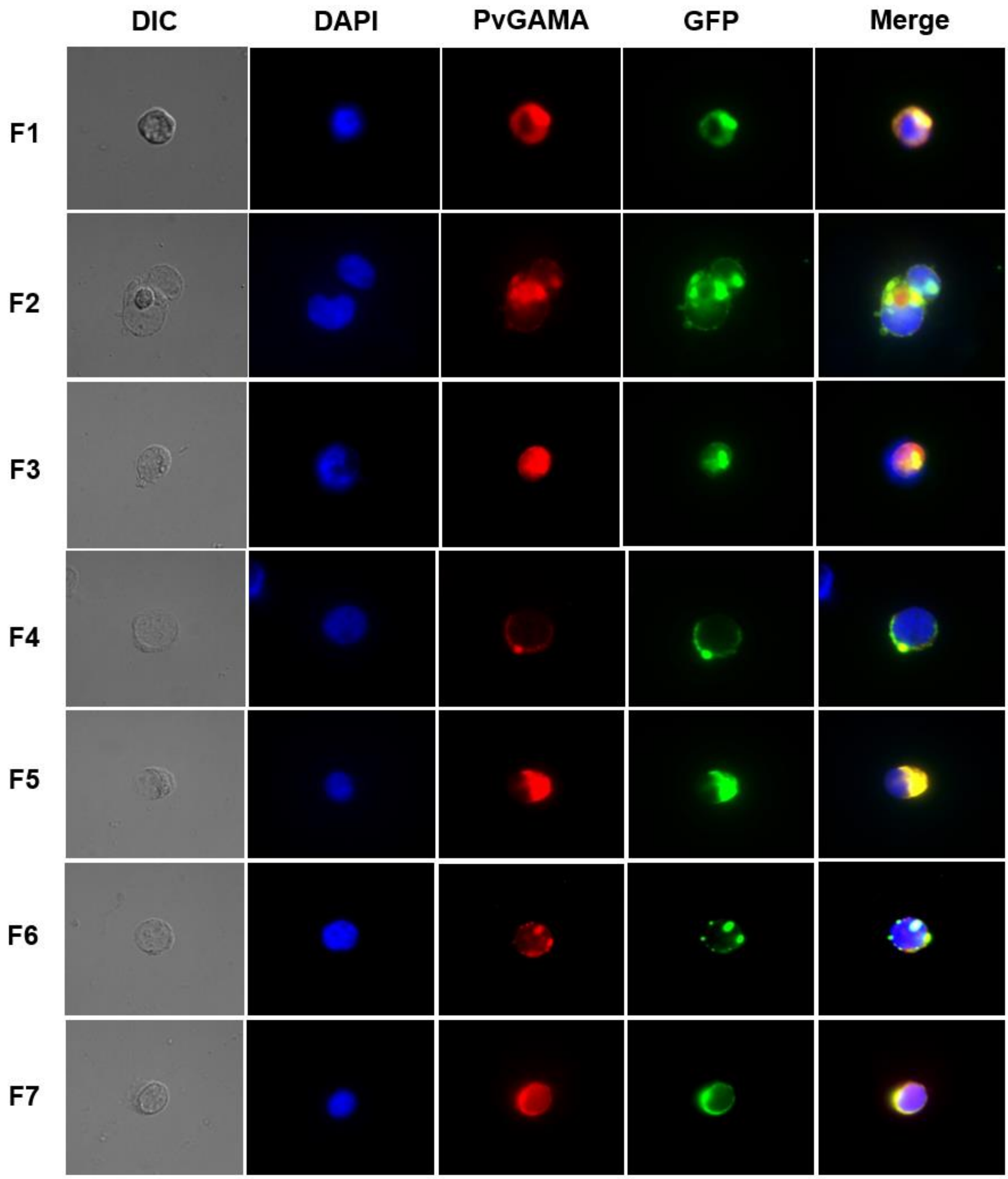

Supplementary Figure S2. Transfection induced expression of PvDBPII and PvGAMA fragments on the surface of HEK 293T cells. Expression of PvDBPII and each fragment of PvGAMA on HEK 293T cells transfected with pEGFP-HSVgD1_PvDBPII, and _PvGAMA-F1 to -F7 (Fig. 1a) plasmid DNA were detected by IFA. Green fluorescent protein (GFP) (green, control), Alexa Fluor 568-conjugated goat anti-rabbit antibody (red, PvGAMA fragments) were used and visualized by confocal microscopy. Rabbit antisera against Phosphate-buffered saline (PBS) was used as negative control.
